# Biomarker quantification by multiplexed quantum dot technology for predicting lymph node metastasis and prognosis in head and neck cancer

**DOI:** 10.18632/oncotarget.9225

**Published:** 2016-05-09

**Authors:** Zhongliang Hu, Guoqing Qian, Susan Müller, Jing Xu, Nabil F Saba, Sungjin Kim, Zhengjia Chen, Ning Jiang, Dongsheng Wang, Hongzheng Zhang, Kristin Lane, Clifford Hoyt, Dong M Shin, Zhuo Georgia Chen

**Affiliations:** ^1^ Department of Hematology and Medical Oncology, Winship Cancer Institute, Emory University School of Medicine, Atlanta, GA, USA; ^2^ Department of Pathology, Xiangya Hospital, Department of Pathology, Xiangya Medical School, Central South University, Changsha, Hunan, China; ^3^ Department of Otolaryngology, Emory University School of Medicine, Atlanta, GA, USA; ^4^ Department of Otolaryngology, Xiangya Hospital, Central South University, Changsha, Hunan, China; ^5^ Biostatistics and Bioinformatics Shared Resource at Winship Cancer Institute, Emory University, Atlanta, GA, USA; ^6^ Department of Biostatistics and Bioinformatics, Emory University School of Public Health, Atlanta, GA, USA; ^7^ Caliper/Perkin Elmer Life Sciences and Technology, Hopkinton, MA, USA

**Keywords:** multiplexed quantum dot, head and neck squamous cell carcinoma, EGFR, E-cadherin

## Abstract

**Purpose:**

To predict lymph node metastasis and prognosis in head and neck squamous cell carcinoma (HNSCC).

**Results:**

The combination of membranous E-cadherin and membranous epidermal growth factor receptor (EGFR) quantified by QD technology with age, gender, and grade had greater predictive power than any of the single biomarkers or the two combined biomarkers quantified by conventional immunohistochemistry (IHC). The predictive power of this model was validated in another independent sample set; the predictive sensitivity of this model for LNM was 87.5%, with specificity up to 97.4%, and accuracy 92.9%. Furthermore, a higher membranous E-cadherin level was significantly correlated with better overall and disease-free survival (OS, DFS; *P* = 0.002, 0.033, respectively), while lower cytoplasmic vimentin and membranous EGFR levels were significantly correlated with better OS (*P* = 0.016 and 0.021, respectively). The combined biomarkers showed a stronger prognostic value for OS and DFS than any of the single biomarkers.

**Methods:**

Multiplexed quantum dots (QDs) were used to simultaneously label E-cadherin, vimentin, and EGFR with β-actin as an internal control. Primary tissue samples from 97 HNSCC patients, 49 with and 48 without LNM were included in the training set. Levels of membranous E-cadherin, cytoplasmic vimentin, and membranous EGFR were quantified by InForm software and correlated with clinical characteristics.

**Conclusions:**

Multiplexed subcellular QD quantification of EGFR and E-cadherin is a potential strategy for the prediction of LNM, DFS, and OS of HNSCC patients.

## INTRODUCTION

Head and neck squamous cell carcinoma (HNSCC) is the sixth most common malignancy worldwide [1]. Despite advances in understanding the molecular mechanisms of HNSCC along with improved diagnosis, the 5-year survival rate has remained virtually unchanged in the past 30 years [2, 3]. Lymph node metastasis (LNM) is significantly associated with poor prognosis in HNSCC. The presence of a single ipsilateral or contralateral metastatic node reduces survival by 50% and bilateral disease by a further 50% [4]. Therefore, the identification of biomarkers associated with LNM could predict tumor behavior and guide treatment of HNSCC.

In one of our previous studies, we demonstrated that the subcellular localization of E-cadherin and epidermal growth factor receptor (EGFR) correlates with LNM of HNSCC [5]. It is known that epithelial-to-mesenchymal transition (EMT) is characterized by diminished epithelial features (such as loss of E-cadherin) and enhanced mesenchymal attributes (such as increased expression of cytoplasmic vimentin, fibronectin, and proteolytic enzymes). EMT has been described in embryologic morphogenesis, fibrosis and recently in tumor invasion and metastatic spread [6, 7]. Loss of E-cadherin expression, leading to reduced intercellular adhesion, is a distinctive event in EMT and is common in metastatic carcinomas [6, 8, 9]. E-cadherin expression in HNSCC tissue specimens has been reported in several studies and is correlated with tumor progression and metastasis [5, 10–13]. Cytoplasmic vimentin is considered a hallmark of mesenchymal-like conversion of epithelial cells and appears to be one of the best indicators of EMT in carcinomas including HNSCC [14–16]. EGFR is another important protein involved in progression of HNSCC. Overexpression of EGFR is observed in 80–90% of HNSCC specimens, and has been associated with a worse clinical outcome [17–20]. Therefore, these three proteins were selected as predictive biomarkers for LNM of HNSCC.

Quantum dots (QDs) are nanoscale particles made from inorganic semi-conductors. QDs have superior signal brightness, photostability, longer excited-state lifetimes, and optimized signal-to-background ratios compared with organic dyes [21, 22]. Furthermore, they have a long excitation and narrow emission spectra and can be excited simultaneously through one appropriate excitation source. Moreover, quantum dot staining is more sensitive and objective than conventional immunohistochemistry [23]. Therefore, QDs are ideal probes for both visualizing and quantifying multiple biomarkers simultaneously in the same sample.

E-cadherin, vimentin, and EGFR have all been reported to be correlated with LNM in HNSCC; however, when using one of these as a single protein marker, it is difficult to achieve both high specificity and sensitivity in the prediction of HNSCC LNM. After establishing a multiplexed QDs in head and neck cancer cell lines [24], in this study, we attempted to predict LNM using multiplexed detection of subcellular levels of the three proteins by QD technology in primary HNSCC samples. Furthermore, to minimize the effect of tissue quality on quantification of QD signals, β-actin was used as an internal control for the first time in a multiplexing system. To our knowledge, this is the first study to predict LNM using EMT-associated markers combined with EGFR using a multiplexed QD-based strategy.

## RESULTS

### Expression of membranous E-cadherin, cytoplasmic vimentin, and membranous EGFR in tumor samples

We initially studied a training set of primary HNSCC samples (*n* = 97). When EGFR is activated or E-cadherin is inactivated, the most obvious changes occur on the tumor cell membrane, while cytoplasmic vimentin is one of the best indicators of EMT in HNSCC. Thus, we selected membranous E-cadherin, membranous EGFR and cytoplasmic vimentin for the analysis. The levels of membranous E-cadherin were lower in tumor samples from patients with LNM [median (range) 3.54 (0.96–7.91) vs. 4.47 (1.19–21.05), *P* = 0.002]; while the levels of cytoplasmic vimentin and membranous EGFR were higher in samples from patients with LNM than from those without LNM [median (range) 13.51 (6.19–34.96) vs. 10.35 (4.5–21.99), *P* < 0.001; 13.74 (5.17–35.32) vs. 7.28 3.88–19.22), *P* < 0.001, respectively] (Table 1). Moreover, tumor samples from patients with LNM showed a lower degree of differentiation than those without LNM (*P* = 0.004).

**Table 1:** Patient characteristics and comparison of biomarker levels between patients with and without LNM

Characteristic	Level	Lymph Node Metastasis		*P*-value^*^
		No *N* = 48	Yes *N* = 49	
Sex	Male	30 (62.5)	32 (65.31)	0.774
	Female	18 (37.5)	17 (34.69)	
Tumor site	Oral cavity	24	26	0.730
	Oropharynx	6	8	
	Larynx	18	15	
Tumor size	T1	15 (31.25)	13 (26.53)	0.337
	T2	19 (39.58)	17 (34.69)	
	T3	9 (18.75)	7 (14.29)	
	T4	5 (10.42)	12 (24.49)	
Differentiation	WD	14 (29.17)	2 (4.08)	0.004
	MD	26 (54.17)	36 (73.47)	
	PD	8 (16.67)	11 (22.45)	
Smoking	No	11 (22.92)	12 (24.49)	0.855
	Yes	37 (77.08)	37 (75.51)	
Age	Mean (± SD)	63.13 (± 10.97)	58.73 (± 12.84)	0.074
Membranous E-cadherin	Median (Range)	4.47 (1.19–21.05)	3.54 (0.96–7.91)	0.002
Cytoplasmic vimentin	Median (Range)	10.35 (4.5–21.99)	13.51 (6.19–34.96)	< .001
Membranous EGFR	Median (Range)	7.28 (3.88–19.22)	13.74 (5.17–35.32)	< .001

* The *p*-value is calculated by chi-square test for sex, tumor stage, differentiation, and smoking; ANOVA for Age; Wilcoxon rank-sum test for membranous E-cadherin, cytoplasmic vimentin, and membranous EGFR.

### Multivariate association of metastasis status with all biomarkers and covariates

In a multivariable model after adjusting for age, gender, grade, tumor stage and the 3 biomarkers in which age, gender and grade were forced, membranous EGFR and membranous E-cadherin were significant independent predictors of LNM, respectively (*P = 0.002 for* E-cadherin and *P* < 0.001 for EGFR) (Table 2).

**Table 2:** Best predictive model of metastasis status of patients after adjusting for 3 biomarkers and age, gender and grade (age, gender and grade were forced in the model)

Covariate	Level	Metastasis = Yes				
		Odds Ratio	95% CI Low	95% CI Up	OR *P*-value	Type3 *P*-value
Age		0.98	0.93	1.03	0.333	0.333
Gender	Male	1.25	0.36	4.30	0.728	0.728
	Female	–	–	–	–	
Grade	WD	0.10	0.01	1.03	0.053	0.104
	MD	0.84	0.19	3.72	0.820	
	PD	–	–	–	–	
Membrane E-cadherin		0.53	0.36	0.79	0.002	0.002
Membrane EGFR		1.41	1.19	1.68	< .001	< .001

Backward selection with an alpha level of removal of .1 was used. Age, gender, and grade were forced in the model. Vimentin was removed from the model.

Hosmer and Lemeshow Goodness-of-Fit Test statistic = 4.938; *P*-value = 0.764 (fitted model is an adequate model).

Likelihood Ratio test statistic = 64.52; *P*-value < 0.001 (The overall logistic regression model was significant). AUC (area under the Receiver Operating Characteristic (ROC) curve) = 0.919; *P*-value < 0.001.

### ROC analysis of predictive value of biomarkers for LNM

**Figure 1:**
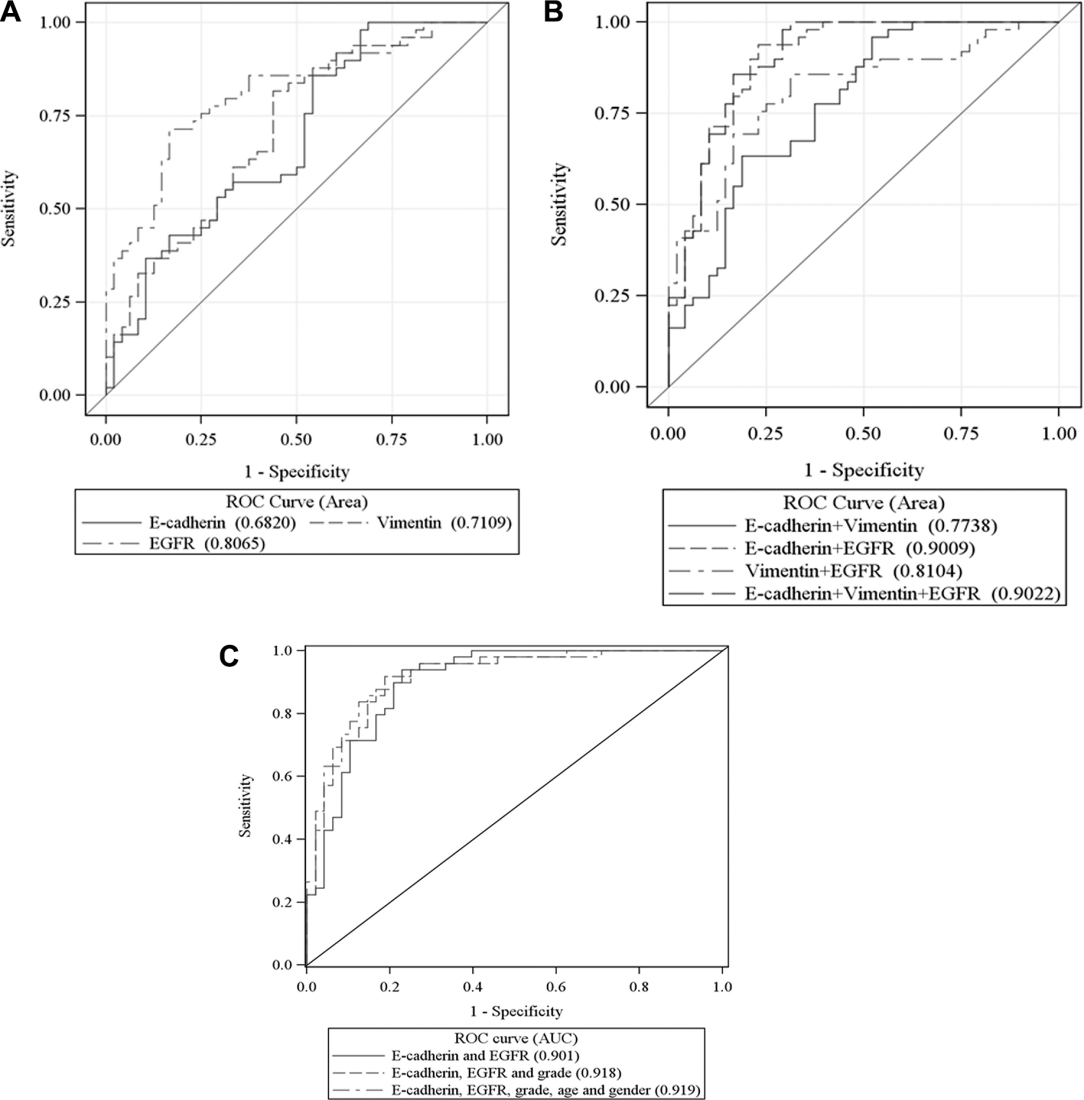
ROC curves for each of the 3 biomarkers (A), combined biomarkers (B), and combined biomarkers and forced age, gender and grade (C) with AUC for prediction of patient's metastasis status

To compare the power of LNM prediction using conventional immunohistochemistry (IHC) with that using QD technology, we performed ROC analyses on both previous IHC [5] and the current QD data which were generated from the same patient sample set. As shown in Table 3, for single biomarkers and the combination of biomarkers, QD analysis showed a better predictive power as compared to conventional IHC (Table 3). All 3 biomarkers were observed to have significant predictive discriminatory power for patients' LNM status. Among them, membranous EGFR had the strongest predictive discriminatory power as a single marker (Table 3). As shown in Table 3 and Figure 1, the combination of membranous E-cadherin and membranous EGFR had a strong predictive discrimination power (AUC: 0.9009). A model combining all three biomarkers did not further improve the prediction of LNM (*P* = 0.842), since cytoplasmic vimentin was not a significant predictor of metastasis status in the multivariable model with all 3 biomarkers (Table 2). A model combining age, gender, grade, membranous E-cadherin and membranous EGFR, shown below, displayed the best predictive power (AUC: 0.919).

**Table 3:** ROC analysis of biomarkers in samples from patients with vs. without metastasis

Biomarker	Area under ROC curve	*P*-value *
***IHC Data**^a^*		
*Single Biomarker Model:*		
Membranous EGFR	0.6044	0.078
Membranous E-cadherin	0.6546	0.009
*Two Biomarker Model:*		
Membranous EGFR+ Membranous E-cadherin	0.6522	0.010
**Quantum Dot Data**		
*Single Biomarker Model:*		
Membranous E-cadherin	0.682	< .001
Cytoplasmic vimentin	0.7109	< .001
Membranous EGFR	0.8065	< .001
*Two Biomarker Model:*		
Membranous E-cadherin + cytoplasmic vimentin	0.7738	< .001
Membranous E-cadherin + membranous EGFR	0.9009	< .001
Cytoplasmic vimentin + membranous EGFR	0.8104	< .001
*Three Biomarker Model:*		
Membranous E-cadherin + cytoplasmic vimentin + membranous EGFR	0.9022	< .001
*Two Biomarker +Age+Gender+Grade Model:*		
Membranous E-cadherin + membranous EGFR +*Age+Gender+Grade*	0.919	< .001
**Area under ROC curves is different from 0.5.		

a IHC data was analyzed from our publication as in reference 5. Quantum dot data was analyzed from the same specimens as in IHC data.

$$\overset{⌢}{p}=\frac{\exp\left(-0.0074-0.0253\times \text{Age}+0.1098\times \text{SexMale}+0.649\times \text{Grade}\_MD-1.4703\times \text{Grade}\_WD-0.6291\times \text{Ecadherin}+0.3465\times EGFR\right)}{1+\text{exp}\left(-0.0074-0.0253\times \text{Age}+0.1098\times \text{SexMale}+0.649\times \text{Grade}\_MD-1.4703\times \text{Grade}\_WD-0.6291\times \text{Ecadherin}+0.3465\times EGFR\right)}$$

In this model, the maximized sum of sensitivity and specificity was 83.7% and 87.5%, respectively, for LNM prediction when using the training set of samples (Table S1).

### Validation of the predictive probability model of metastasis status

To verify the predictive probability model of metastasis status consisting of age, gender, grade, membranous E-cadherin and membranous EGFR, another independent sample set was used. As shown in Table 4, the predictive sensitivity of this model was 87.5%, with specificity up to 97.4%, and accuracy 92.9%.

**Table 4:** Validation of the model for lymph node metastasis prediction

	Met	Non-Met	*P*-value	Sensitivity (%)	Specificity (%)	Accuracy (%)
Prediction-Met	28	1	< 0.0001	87.5	97.4	92.9
Prediction-Non-Met	4	37				

### Univariate survival analysis of DFS and OS

Patients with LNM had a higher risk of death compared to those without LNM (*P* = 0.002). Older patients also had a higher risk of death (*P* = 0.01). A higher level of membranous E-cadherin was significantly correlated with better OS (*P* = 0.002), while lower levels of cytoplasmic vimentin and membranous EGFR were significantly associated with better OS (*P* = 0.016 and 0.021, respectively) (Table S2). Patients with LNM had a higher risk of disease progression than those without LNM (*P* = 0.004). A higher level of membranous E-cadherin was significantly associated with a better DFS (*P* = 0.033) (Table S3). All three biomarkers were significantly correlated with OS and DFS after being dichotomized by the optimal cut-off point driven by survival analysis (Figure S1). We observed that all three biomarkers together showed a stronger prognostic value for OS and DFS than any single biomarker.

### Multivariable survival analysis of DFS and OS with all biomarkers and covariates

In a multivariable model after adjusting for age, differentiation, sex, smoking history, tumor stage, LNM status and the 3 biomarkers, cytoplasmic vimentin, age, and LNM status were identified as significant predictors of OS (*P* = 0.033, < 0.001, and 0.018, respectively). Membranous E-cadherin was marginally significantly related to OS (*P* = 0.056) (Table S4). In a multivariable model after adjusting for age, differentiation, sex, smoking, tumor stage, LNM status and the 3 biomarkers, LNM status was significantly associated with DFS (*P* = 0.002) and age was marginally significantly related to DFS (*P* = 0.071) (Table S5).

## DISCUSSION

Accurately predicting LNM and overall prognosis in HNSCC can provide useful information for the optimization of treatment plans for patients with HNSCC as well as furthering our understanding of the biology underlying metastases. Currently, there is no standardized and consistent method to predict LNM of HNSCC using multiple protein biomarkers. In this study, we used selected EMT-related protein markers objectively quantified by QD technology to predict LNM and prognosis of HNSCC. Using a training set of HNSCC tissue samples, we initially established a LNM prediction model consisting of age, gender, grade, membranous EGFR and membranous E-cadherin. As expected, excellent levels of sensitivity (87.5%) and specificity (97.4%) were observed in the validation sample using the same model.

Using nanoparticle QD-based IHF, we were able to not only simultaneously quantify multiplexed biomarker levels in the same cell, but also detect their levels at specific subcellular locations. Moreover, QD-IHF can reveal population characteristics, such as analyte range, distribution, and variance among cells. Our study found less membranous E-cadherin, more cytoplasmic vimentin, and more membranous EGFR in primary tumor samples with LNM than in those without LNM. All three biomarkers had significant power for predicting LNM. The combination of membranous E-cadherin and membranous EGFR had stronger power to predict LNM than other combinations. Although IHC can semiquantify the subcellular levels of EGFR and E-cadherin, the LNM prediction power using IHC is much lower than using QD technology as demonstrated in the ROC analysis. Furthermore, membranous E-cadherin and EGFR together showed a stronger prognostic value for OS than any of the single biomarkers. Comparing these findings with those of our previous IHC-based study of EGFR and E-cadherin [5], QD-based IHF analysis is more objective and powerful, therefore, more accurate for prognosis of the disease. Since many proteins including EGFR and E-cadherin demonstrate dramatic changes in membrane expression when they are involved in biological activities, it is ideal to quantify the subcellular levels, rather than total, levels of these proteins. Another advantage of QD technology is that the multiplexed quantification of QD signals makes it easier to detect the staining signals of more than one protein from the nucleus, cytoplasm, or membrane of the same cell. Thus, QD-based IHF is a novel technology that uniquely addresses such issues in HNSCC biomarker studies. For QD staining, we used β-actin as an internal control for tissue quality because, as a housekeeping protein, it is constitutively expressed in tissues and we therefore quantified the biomarker levels as a value relative to β-actin levels. In our study, 3 samples were excluded because their β-actin expression was not detected. We also tested another housekeeping protein, glyceraldehyde 3-phosphate dehydrogenase (GAPDH), as a tissue quality control marker. The QD signal from GAPDH was well correlated with that from β-actin (Figure S2), suggesting that tissue quality may have a similar effect on immunostaining of any housekeeping protein which can serve as the internal control.

HPV status has been correlated with LNM [25, 26]. However, our sample set, particularly the non-metastatic group, contained few oropharyngeal tissues, which limited the analysis of HPV status as a confounding variable. Therefore, we did not consider the expression of p16, a surrogate of high-risk HPV status in the current study. Furthermore, because our sample set included a limited number of patients with advanced HNSCC, tumor size was not observed to be significantly associated with prognosis in univariate analysis.

In summary, the subcellular expression levels of multiplexed biomarkers were assessed for the prediction of LNM using QD-based IHF technology. We found that the combination of membranous EGFR and membranous E-cadherin demonstrated strong predictive power for LNM and improved prognosis as compared to single biomarkers. Our study also demonstrated that the detection of multiplexed biomarkers using QD technology can serve as a stronger prognostic tool than a single biomarker. This method can have distinct advantages when tissue sample sizes are limited as multiple markers can be assessed simultaneously. This is a proof of principle study to demonstrate the potential use of QD-based biomarker multiplexing to establish a predictive model for LNM in head and neck cancer. Further optimization and validation of our model using prospectively recruited tumor samples is warranted in large-scale studies and the inclusion of biomarkers in combination with relevant clinical factors such as HPV status may result in establishment of a standardized method for the prediction of LNM and prognosis of HNSCC.

## MATERIALS AND METHODS

### Patient samples

Using an Institutional Review Board–approved consent for tissue acquisition, clinical samples for this study were obtained from surgical specimens from patients diagnosed with HNSCC from 1994 to 2003 at the Winship Cancer Institute of Emory University (Atlanta, GA). The primary treatment for these patients was surgery and no prior treatment with radiation and/or chemotherapy was administered. Patient samples consisted of a training set to establish an optimized LNM predictive model and a validation set. The training set comprised primary SCC samples from 49 patients with LNM and 48 patients without LNM, whereas the validation set consisted of 70 samples including 32 cases with LNM, and 38 without LNM. In the non-LNM group, none of the patients developed metastases within 2 years of the initial procedure. The clinical information on the samples was obtained from the surgical pathology reports in the Department of Pathology at Emory University according to the regulations of the Health Insurance Portability and Accountability Act. The clinicopathologic parameters for the training set, including age, sex, tobacco history, and disease stage, are characterized and listed in Table 1. Each patient's overall survival (OS) and disease-free survival (DFS) were documented through June 2012. Samples in the validation set were selected with similar anatomic locations as the training set (data not shown).

In the training set, the number of oropharyngeal samples was limited in both the non-metastasis and metastasis groups (Table 1). Therefore, the contribution of HPV status to metastasis was not studied.

### Quantum dot-based immunohistofluorescence (QD-IHF)

QD-IHF for the measurement of EGFR, E-cadherin, cytoplasmic vimentin, and β-actin in tumor tissues was performed as described previously. Briefly, formalin fixed and paraffin embedded (FFPE) tissue blocks were cut into 4–5 μm sections. After deparaffinization and rehydration, antigen retrieval was performed by heating with citrate buffer (10 mmol/L, pH 6.0) in a microwave for 10 min. The tissue slides were blocked with 10% normal goat serum for 20 min before the primary antibody incubation. Multiple markers were stained initially in a sequential manner with a 3 × 5 min phosphate-buffered saline (PBS) rinse after each step of incubation. The immunoreaction sequences were: 1) primary rabbit anti-human EGFR antibody (Biogenex, Fremont, CA), overnight at 4°C; secondary goat anti-rabbit conjugated-QD705 (Invitrogen, Carlsbad, CA), at 37°C, 2 hour. 2) primary mouse anti-human E-cadherin (BD Pharmingen, San Jose, CA), rabbit anti-human vimentin (Cell Signaling, Danvers, MA), and chicken anti-human β-actin (Novus Biologicals, Littleton, CO) antibodies for 2 hours at room temperature; secondary goat anti-mouse conjugated-QD565, goat anti-chicken conjugated-QD655, and goat anti-rabbit conjugated-QD625 (Invitrogen) antibodies were incubated in a cocktail manner. The slides were washed three times with 1 × PBS, counterstained with 4′,6-diamidino-2-phenylindole (DAPI), mounted, and stored at 4°C in dark conditions. For negative controls, primary antibodies were replaced with isotype- and species-matched control antibodies.

### Image acquisition and unmixing

A CRi spectral imaging system (Caliper/Perkin Elmer Life Sciences and Technology, Hopkinton, MA) with Nuance v3.1 software was used to take multiple images following the manufacturer's protocol. For each sample, a cube file consisting of serial images was acquired at 10 nm wavelength intervals from 425 to 720 nm, a range covering the wavelengths of the active fluorescent QDs. To avoid variations due to cell heterogeneity, five images were randomly taken from cancerous tissue from each tissue specimen for subsequent quantification.

**Figure 2:**
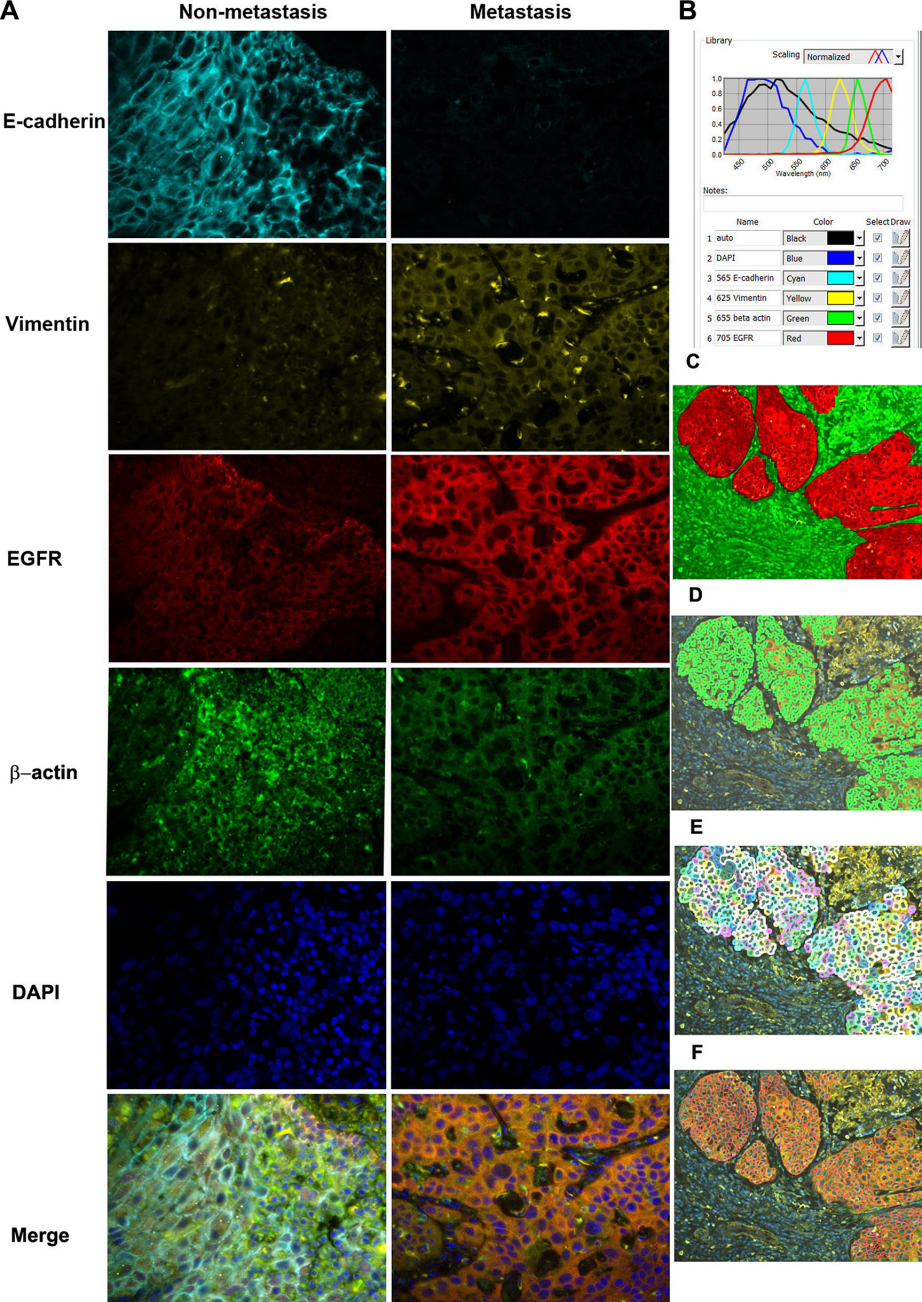
Simultaneous detection of three biomarkers plus control using QD-based IHF system (**A**) library composed of DAPI, auto-fluorescence, QD565, QD625, QD655, and QD705 was initially set for the analysis. Signals in the image cube were unmixed according to their wavelengths in the library and then the corresponding signals were separated. E-cad is shown in cyan, EGFR in red, cytoplasmic vimentin in yellow, and β-actin in green. A: The expression of three biomarkers in primary tumors from patients with or without LNM. (**B**) Quantum dot library (normalized spectrum). C-F: segmentation of cancer cell from stroma (**C**), nucleus (**D**), cytoplasm (**E**), and membrane (**F**).

A spectral library for QDs 565, 625, 655, and 705 nm was built for deconvolution. The spectral library was then used to unmix the cube files. These images represent the distribution of each of the QDs and autofluorescence in the tissue. After the deconvolution of the images, the background signal was filtered away and only the true positive signals were shown on the images, as shown in Figure 2.

### Signal quantification

To extract the individual QD signals, the spectral library was used to unmix the imaging cube using inForm v1.4 software (Caliper/Perkin Elmer), following the manufacturer's protocol. First, a training set comprising two categories of tissue was created: cancer and stroma. The trained tissue segmentation method was used to separate cancer from its surrounding stroma until the accuracy was over 90% and differentiation of the two classes could no longer be improved. Then a built-in algorithm was used to define the membranous, cytoplasmic, and nuclear subcellular regions. Based on an analysis of images at 200× magnifications, QD fluorescence intensity in each cell was exported to an Excel spreadsheet (Microsoft, Seattle, WA) and the relative values of E-cadherin, cytoplasmic vimentin, and EGFR levels in different subcellular regions to β-actin were obtained and subjected to statistical analysis.

### Statistical analysis

Three biomarkers and covariates (gender, tumor stage, differentiation, smoking history, and age) were compared between samples from patients with and without LNM using chi-square test, ANOVA (analysis of variance) or Wilcoxon rank-sum test where appropriate. Each biomarker was analyzed with logistic regression to estimate its effect on the prediction of LNM. A best predictive model was also identified by entering three biomarkers and covariates into a logistic model and using a backward variable selection method with an alpha level of removal of 0.1. The ability of a biomarker to predict LNM status was further determined by using receiver operating characteristic (ROC) curves and measuring the area under the curve (AUC). Whether the AUCs of ROC curves were different from 0.5 (no discrimination ability) was tested with Chi-Square tests. To obtain optimal cut-off points with the best discrimination power for metastasis status, sensitivity and specificity pairs were obtained in the logistic regression under all the possible thresholds. The optimal cut-off point for each biomarker was calculated where the maximum sum of sensitivity and specificity was achieved.

Membranous E-cadherin, cytoplasmic vimentin, and membranous EGFR were further dichotomized by the optimal cut-off point, which corresponds to the most significant relationship with OS or DFS based on the log rank statistic. Cut-off values for membrane E-cadherin, cytoplasmic vimentin, and membrane EGFR were 6.57, 11.92, and 10.93, respectively. Kaplan-Meier survival estimates were then calculated for each group of patients stratified based on the optimal cut-off points along with a log-rank test. Univariate survival analysis for each covariate as well as membranous E-cadherin, cytoplasmic vimentin, and membranous EGFR was carried out using the Cox proportional hazards model. The proportional hazard assumption was also assessed. A multivariable survival analysis of membranous E-cadherin, cytoplasmic vimentin, and membranous EGFR was conducted after adjusting for gender, age, grade, and metastasis status using a backward variable selection method with an alpha level removal of 0.1. All analyses were performed using SAS 9.3 (SAS Institute, Inc., Cary, North Carolina) and the significance level was 0.05.

## SUPPLEMENTARY MATERIALS


