# Supplementary material for: Biomarker quantification by multiplexed quantum dot technology for predicting lymph node metastasis and prognosis in head and neck cancer
Source: Oncotarget. 2016 May 9;7(28):44676–85. doi: 10.18632/oncotarget.9225 (PMC5190127; doi:10.18632/oncotarget.9225)
Supplement: Supplementary file 1 [file oncotarget-07-44676-s001.pdf]

Biomarker quantification by multiplexed quantum dot technology for predicting lymph node metastasis and prognosis in head and neck cancer

Supplementary Materials

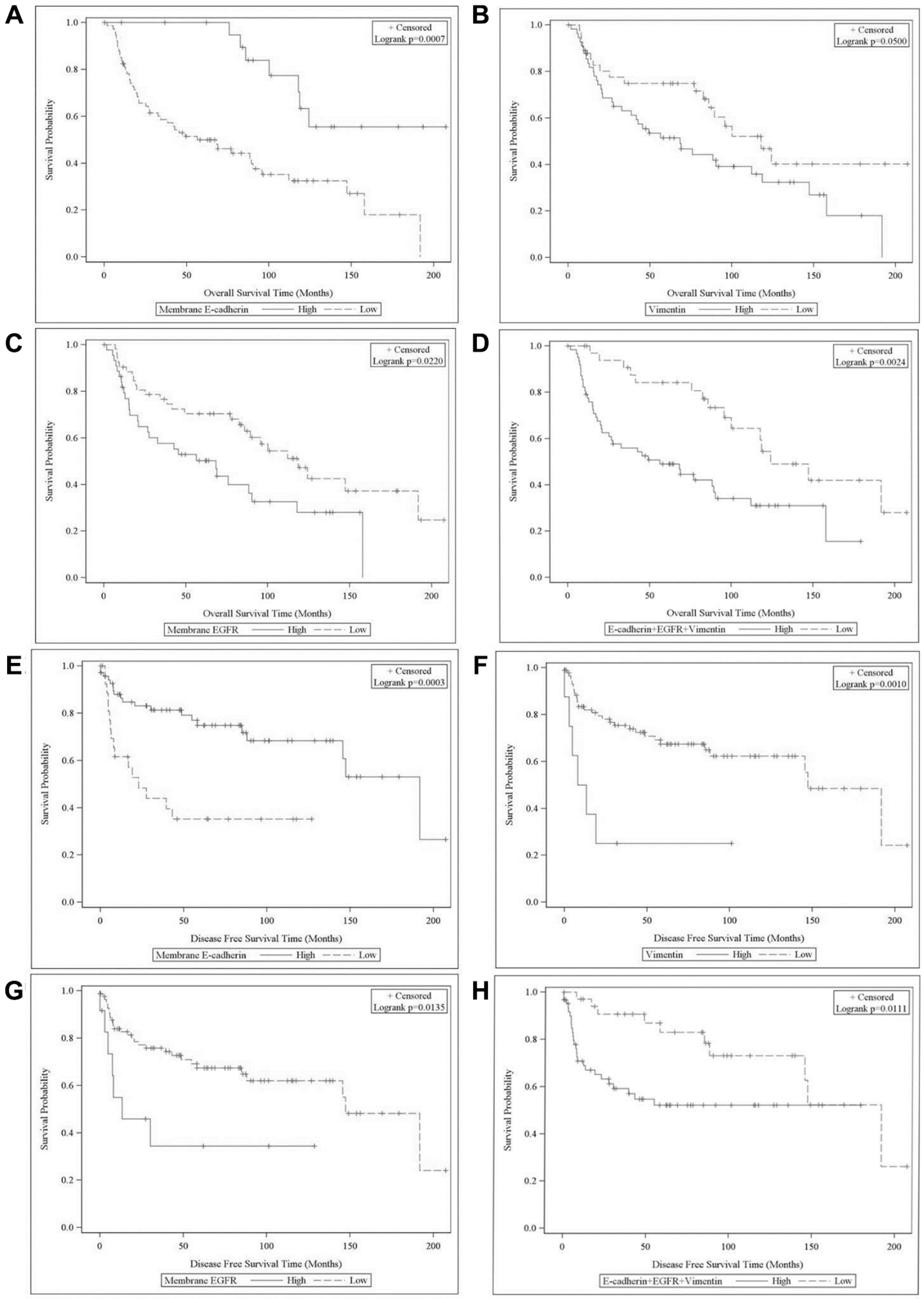

Supplementary Figure S1: KM curves of OS (A to D) and DFS (E to H) for membranous E-cadherin (A and E), cytoplasmic vimentin (B and F), membranous EGFR (C and G), and all three biomarkers (D and H) cut by the optimal cut-point driven by survival analysis.

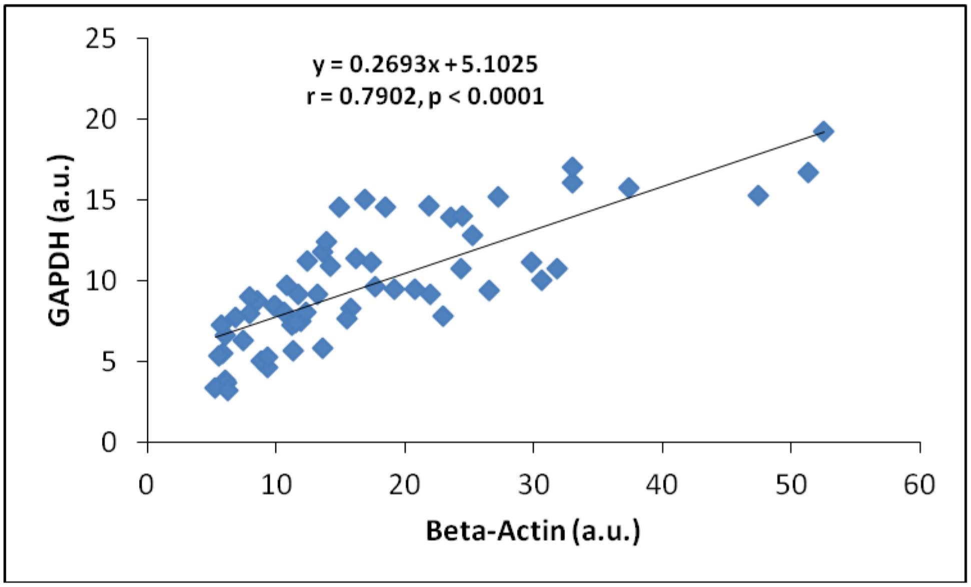

Supplementary Figure S2: Correlation of QD-IF signal (arbitrary units) of GAPDH with that of β-actin. Premixed chicken anti-human β-actin (1:1000) and rabbit anti-human GAPDH (1:500) (Trevigen, Gaithersburg, MD) was incubated with tumor slides at 4°C overnight, followed by incubation with a mixture of goat anti-chicken conjugated-QD655 for β-actin (1:100) and goat anti-rabbit conjugated-QD705 for GAPDH (1:50) (Invitrogen, Carlsbad, CA) for 1 hour at 37°C in a humid environment. Quantification was performed on 60 randomly selected images by CRi spectral imaging system (Caliper/Perkin Elmer Life Sciences and Technology, Hopkinton, MA) with Nuance v3.1 software. Correlation was determined by Pearson correlation using SigmaPlot 12.0, Systat Software Inc, San Jose, CA).

Supplementary Table S1: Summary of cut-off points for different sensitivities and specificities with metastasis vs. no metastasis

Parameter	Sensitivity = 90%	Specificity = 90%	Maximize sum of specificity and sensitivity	≥ 50% to be Metastasis
Model, adjusted for E-cadherin, EGFR, Grade, Age and Gender:				
Cut-off = −0.0253*Age + 0.1098*SexMale + 0.649*Grade_MD −1.4703*Grade_WD −0.6291*MemE-cad + 0.3465*MemEGFR	Specificity = 85.4%<br>Cut-off = 0.246	Sensitivity = 85.7%<br>Cut-off = 0.246	Sensitivity = 83.7%<br>Specificity = 87.5%<br>**Cut-off = 0.320**	**Cut-off ≥ 0.012**

Supplementary Table S2: Univariate OS analysis

Covariate	Level	*N*	Overall Survival Time (Months)<br>Hazard Ratio	95% CI Low	95% CI Up	HR *P*-value	*P*-value
Metastasis	Yes	49	2.365	1.355	4.128	0.002	0.002
	No	48	–	–	–	–	
Sex	Male	62	0.988	0.563	1.733	0.966	0.966
	Female	35	–	–	–	–	
Tumor size	T1	28	0.595	0.273	1.297	0.192	0.478
	T2	36	0.609	0.292	1.270	0.186	
	T3	16	0.585	0.251	1.363	0.214	
	T4	17	–	–	–	–	
Differentiation	MD	62	0.941	0.462	1.917	0.866	0.962
	PD	19	0.878	0.354	2.177	0.779	
	WD	16	–	–	–	–	
Smoking	Yes	74	0.879	0.470	1.643	0.685	0.685
	No	23	–	–	–	–	

Overall Survival Time (Months)							
**Covariate**	**Level**	***N***	**Hazard Ratio**	**95% CI Low**	**95% CI Up**	**HR *P*-value**	***P*-value**
Age		97	1.034	1.008	1.060	0.010	0.010
Membranous E-cadherin		97	0.868	0.791	0.951	0.002	0.002
Cytoplasmic vimentin		97	1.070	1.012	1.130	0.016	0.016
Membranous EGFR		97	1.046	1.007	1.088	0.022	0.021

Supplementary Table S3: Univariate DFS analysis

Disease Free Survival Time (Months)							
**Covariate**	**Level**	***N***	**Hazard Ratio**	**95% CI Low**	**95% CI Up**	**HR *P*–value**	***P*–value**
Metastasis	Yes	49	2.699	1.339	5.439	0.005	0.004
	No	48	–	–	–	–	
Sex	Male	62	0.865	0.440	1.698	0.673	0.672
	Female	35	–	–	–	–	
Tumor size	1	28	1.117	0.408	3.059	0.830	0.411
	2	36	0.945	0.348	2.567	0.912	
	3	16	0.389	0.097	1.563	0.183	
	4	17	–	–	–	–	
Differentiation	MD	62	1.192	0.484	2.937	0.702	0.836
	PD	19	0.935	0.301	2.906	0.907	
	WD	16	–	–	–	–	
Smoking	Yes	74	0.944	0.427	2.087	0.887	0.887
	No	23	–	–	–	–	
Age		97	1.016	0.986	1.046	0.296	0.296
Membranous E-cadherin		97	0.892	0.801	0.994	0.038	0.033
Cytoplasmic vimentin		97	1.056	0.987	1.130	0.117	0.117
Membranous EGFR		97	1.034	0.986	1.084	0.173	0.171

Supplementary Table S4: Multivariable predictive model of OS

Overall Survival Time (Months)						
**Covariate**	**Level**	**Hazard Ratio**	**95% CI Low**	**95% CI Up**	**HR *P*–value**	***P*–value**
Membranous E-cadherin		0.904	0.816	1.002	0.056	0.056
Cytoplasmic vimentin		1.067	1.005	1.133	0.033	0.033
Age		1.048	1.020	1.076	< .001	< .001

Overall Survival Time (Months)						
**Covariate**	**Level**	**Hazard Ratio**	**95% CI Low**	**95% CI Up**	**HR *P*–value**	***P*–value**
Metastasis	Yes	2.120	1.135	3.961	0.018	0.018
	No	–	–	–	–	

Backward selection with an alpha level of removal at .1 was used. Differentiation, membranous EGFR, Sex, Smoking, and tumor stage were removed from the model.

Supplementary Table S5: Multivariable predictive model of DFS

		Disease Free Survival Time (Months)				
**Covariate**	**Level**	**Hazard Ratio**	**95%CI Low**	**95%CI Up**	**HR *P*–value**	***P*–value**
Age		1.027	0.998	1.057	0.071	0.071
Metastasis	Yes	3.137	1.527	6.445	0.002	0.002
	No	–	–	–	–	

Backward selection with an alpha level of removal at .1 was used. Differentiation, membranous EGFR, membranous E-cadherin, Sex, Smoking, tumor stage, and cytoplasmic vimentin were removed from the model.
